# Inosine Improves Functional Recovery and Cell Morphology Following Compressive Spinal Cord Injury in Mice

**DOI:** 10.1089/neur.2024.0081

**Published:** 2024-10-02

**Authors:** Ricardo Cardoso, Fellipe Soares dos Santos Cardoso, Bruna dos Santos Ramalho, Guilherme dos Santos Maria, Roberta Ramos Cavalcanti, Tiago Bastos Taboada, Juliana Silva de Almeida, Ana Maria Blanco Martinez, Fernanda Martins de Almeida

**Affiliations:** ^1^ Laboratório de Neurodegeneração e Reparo—Departamento de Anatomia, Patológica—Hospital Universitário Clementino Fraga Filho/UFRJ, Rio de Janeiro, Brazil.; ^2^ Instituto de Ciências Biomédicas—ICB/UFRJ, Rio de Janeiro, Brazil.

**Keywords:** axonal regeneration, inosine and functional recovery, spinal cord injury (SCI)

## Abstract

Spinal cord injury (SCI) is one of the most serious conditions of the central nervous system, causing motor and sensory deficits that lead to a significant impairment in the quality of life. Previous studies have indicated that inosine can promote regeneration after SCI. Here we investigated the effects of inosine on the behavioral and morphological recovery after a compressive injury. Adult female C57BL/6 mice were subjected to laminectomy and spinal cord compression using a vascular clip. Inosine or saline injections were administered intraperitoneally, with the first dose performed 24 h after injury and daily for 7 days after injury. The mice were evaluated using Basso Mouse Scale (BMS), locomotor rating scale, and pinprick test for 8 weeks. At the end, the animals were anesthetized and euthanized, and the spinal cords were collected for morphological evaluation. Inosine-treated animals presented better results in the immunostaining for oligodendrocytes and in the number of myelinated fibers through semithin sections compared to saline-treated animals, showing that there was a greater preservation of the white matter. Analysis of the immunoreactivity of astrocytes and evaluation of the inflammatory profile with macrophage labeling revealed that the animals of the inosine group had a lower immunoreactivity when compared to control, which suggests a reduction of the glial scar and less inflammation, respectively, leading to a more favorable microenvironment for spinal cord regeneration. Indeed, inosine-treated animals scored higher on the BMS scale and presented better results on the pinprick test, indicating that the treatment contributed to motor and sensory recovery. After the animals were sacrificed, we obtained the electroneuromyography, where the inosine group showed a greater amplitude of the compound muscle action potential. These results indicate that inosine contributed to the regeneration process in the spinal cord of mice submitted to compressive injury and should be further investigated as a candidate for SCI therapy.

## Introduction

Traumatic spinal cord injury (SCI) is one of the most common and aggressive conditions of the central nervous system (CNS) and usually leads to functional permanent disabilities with devastating impacts on the quality of life.^1,2^ The prevalence of SCI ranges between 10.4 and 83 per million people per year; 90% result from trauma.^3,4^

SCI triggers neuron and glial cell death that is followed by some important events in the distal stump of damaged axons, known as Wallerian degeneration, which is characterized by an abrupt and asynchronous axonal collapse. This is due to calpain activation caused by an ionic imbalance of Ca^2+^ and energetic depletion.^5–8^ After the primary events on the injured spinal cord, a cascade of secondary events starts with the breakdown of the blood–brain barrier, allowing infiltration of inflammatory cells, vascular damage, edema and subsequent ischemia, excitotoxicity by glutamate release, and formation of free radicals. All these secondary events aggravate the lesion core and increase neuron and glial cell death.^9–14^ In addition to Wallerian degeneration, a large area of demyelination, due to oligodendrocyte death and myelin breakdown, is also observed following SCI.^15,16^

A harmful microenvironment and permanent inflammation after injury create an adverse scenario for CNS axon.^3,13^ The regenerating proximal stump lies in an inhibitory environment that is formed by the glial scar and has myelin debris.^3,5,17,18^ Glial scar, composed of reactive astrocytes and inflammatory cells, surrounds many cavities that are formed after trauma, acting as a physical barrier against regenerating axons. In addition, reactive astrocytes fill these cavities with chondroitin sulfate proteoglycans, which added to myelin debris OMGp (oligodendrocyte myelin glycoprotein), MAG (myelin-associated glycoprotein), and NOGO and activate the RhoA/ROCK pathway, leading to growth cone collapse by inhibiting axon elongation.^18,19^ Due to these events, regeneration of the CNS is considered abortive.^13^

In the field of molecular therapies for promoting regeneration and neuroprotection, inosine, a purine derived from the deaminating of adenosine, emerges as a promising treatment for SCI.^5,20^ An *in vitro* study was conducted using dissociated cultures of retinal ganglion cells treated with inosine. The treatment stimulated axonal growth through the upregulation of GAP 43.^21^ A similar upregulation of GAP 43 and axonal growth was observed after cortical spinal tract (CST) injury and inosine treatment.^22^ After hemisecting of spinal cord and consequent CST injury, inosine promoted axon sprouting and promoted corticospinal-LPSN synapses, leading to functional recovery.^23^ To investigate the neuroprotective effects of inosine, an *in vitro* study used glial cell cultures submitted to glucose deprivation and mitochondrial respiratory chain inhibition with amobarbital, revealing that inosine treatment promoted glial cell viability and survival.^24^ The neuroprotective effect was also observed on oral administration of inosine after SCI insufflated the balloon of a Fogarty catheter inserted at epidural space. The animals that took inosine presented a higher rate of survival neurons and recovery of motor function and bladder activity if compared to controls.^25^ Inosine can also promote the reduction of macrophage/microglia around the lesion core^11^ and also attenuate the secondary events.^11,26^

Taking into consideration the severity of SCI and the promising effects of inosine, we here investigated the neuroprotection potential of inosine in SCI regeneration in a mouse model.

## Materials and Methods

### Surgical procedure

Female C57Bl/6 mice, 8–10 weeks old (*n* = 24), were anesthetized with ketamine (15 mg/kg) and xylazine (100 mg/kg) by intraperitoneal injection and subjected to compression injury for 1 min at the T9 level using a 30-g vascular clip (Kent Scientific Corporation, INS14120, USA), as previously described by Marques and colleagues.^15^ After surgery, the animals were allowed to recover on a warm pad and received 1 mL of saline solution to rehydrate. The bladders were manually expressed twice a day until spontaneous urinary function recovery. All our experiment procedures underwent evaluation and received approval from the Ethics Committee for Animal Research at the Federal University of Rio de Janeiro (CEUA-UFRJ, MACAE19).

### Treatment and experimental groups

After SCI, the animals were randomly divided into two groups: saline and inosine. The saline and inosine (260 mM ∼70 mg/day/kg body weight, SIGMA)^23^ animals were injected intraperitoneally, 24 h after injury and daily until the seventh-day post-injury.

### Immunohistochemistry

Eight weeks after injury, we performed immunohistochemistry for glial fibrillary acidic protein (GFAP), an astrocyte marker, Gal-C, an oligodendrocyte marker, F4/80, a macrophage marker, and adenosine 2A receptor (A2AR), a P1 adenosine receptor marker. Animals (three per group) were transcardially perfused with 4% paraformaldehyde (PFA) in 0.1 M phosphate buffer (pH 7.4). After dissection and post-fixation, the segments of the spinal cord were divided into rostral and caudal to the lesion epicenter, cryoprotected in increasing concentrations of sucrose solution up to 30% and left in this solution overnight and then ice-cold embedded in OCT (Optimal Cutting temperature, Tissue Tek), using liquid nitrogen. A total of 10 µm thick sections were obtained with a cryostat and collected on gelatin-coated glass slides. The slides were then incubated in 0.06% potassium permanganate for 15 min to avoid spinal cord autofluorescence, washed in PBS, incubated with a blocking solution containing 10% NGS in PBS and 0.3% Triton for 1 h at room temperature, washed in PBS 0.3% triton, and incubated in primary antibody rabbit anti-GFAP (1:200; Sigma-Aldrich), rat anti-Gal-C (1:100; Sigma-Aldrich), rat anti-F4/80 (AbD Serotec, 1:200), and rabbit anti-A2AR (1:200, Invitrogen) overnight. On the next day, the slides were washed in 0.3% PBS triton, incubated with secondary antibody Alexa 546 goat anti-rabbit (Invitrogen, 1:500), Alexa 546 goat anti-rat (Invitrogen, 1:500), and Alexa 488 goat anti-rat (Invitrogen, 1:500) for 1:30 h, followed by three washes of 10 min each, counterstained with nuclear label DAPI (4′,6-diamidino-2-phenylindole, Molecular Probes, USA, 1:10,000), and coverslipped with Fluoromount (Sigma-Aldrich). Primary antibodies were omitted for negative controls. The sections were observed under a Zeiss Axioskop 2 Plus fluorescence microscope, equipped with a Zeiss Axiocam MRC camera, and under a Zeiss Cell Discoverer 7, confocal microscope. The immunolabeled area was quantified using the Image-Pro-Plus software.

### Semithin sections

Eight weeks after treatment, animals (*n* = 3 per group) were anesthetized with ketamine and xylazine as described above and perfused intracardially with a solution of 4% PFA and 1% glutaraldehyde in 0.1 M phosphate buffer (pH 7.4). The spinal cords were extracted, divided into three different segments (the epicenter of the lesion and rostral and caudal to it), and post-fixed by immersion for 6 h in a solution of 1% osmium tetroxide containing 0.9% potassium ferrocyanide in 0.1 M cacodylate buffer, at room temperature. Samples were washed three times with 0.1 M phosphate buffer (pH 7.4), dehydrated in a graded acetone series, embedded in resin (Embed-812, SEM), and polymerized for 48 h at 60°C. Semithin (500 nm) sections from each group were obtained on an RMC ultramicrotome. The semithin sections were stained with toluidine blue and observed under a Zeiss microscope (Axioscop 2 Plus), and the pictures, with a magnification of 400x and 1000x, were acquired using the Axiovision Program version 4.5 (Zeiss).

### Myelinated fibers counting and morphometric evaluation

To analyze the number of myelinated nerve fibers and morphometric evaluation, we used 400x and 1000x magnification images, respectively. The myelinated fibers were counted by two blind observers using the Image J software. For morphometric evaluation, we measured the axon area, myelin area, and fiber area and calculated the *g*-ratio values in six images from the right and left sides representing the anterior, lateral, and posterior funiculus, using the Image J software. The *g*-ratio was calculated by dividing the inner axonal diameter by the outer fiber diameter, and results were separated in ranges of 0.1–0.2, 0.2–0.3, 0.3–0.4, 0.4–0.5, 0.5–0.6, 0.6–0.7, 0.7–0.8, 0.8–0.9, and 0.9–1.0. In spinal cords, the ideal *g*-ratio is around the 0.8 range.^27^

### Electroneuromyography

To perform a functional study of the nerve regeneration 8 weeks after SCI, animals were anesthetized with ketamine (100 mg/kg) and xylazine (15 mg/kg). Then the spinal cord at C0–C1 was exposed, along with the gastrocnemius muscle and its corresponding tendon, the calcaneus. Using the Power Lab 4/35 device (AD Instruments, Germany), an electrical stimulus with an intensity of 10 mV was triggered on the spinal cord at C1 level through a hooked bipolar electrode, with the cathode distant 2 mm from the anode. To record the compound muscle action potential (CMAP), three-needle electrodes were used. The active electrode was inserted at the gastrocnemius, the reference electrode at the calcaneus tendon, and the ground electrode placed under the skin at the base of the animal tail. The amplitude (mV) of the CMAP was analyzed using the LabChart 8 software (AD Instruments, Germany).

### Behavioral tests

To evaluate the recovery of motor and sensory functions, animals were evaluated before and on the following day of the injury and weekly up to 8 weeks after treatment. For motor function, we performed the Basso Mouse Scale (BMS),^28^ a 9-point scale that evaluates the locomotor ability and locomotion features such as ankle movement, paw position, weight support, plantar steps, hindlimb and forelimb coordination, and trunk stability. For sensory function, we performed an adaptation of the pinprick test used on sciatic nerve injuries, described by Ma and coworkers^29^ and previously used and validated for CNS injuries by Ramalho and coworkers^30^ (see for further information).

### Statistical analyses

All statistical analyses were performed using GraphPad Prism 8 (GraphPad software, USA). We used the *t-*test, two-tailed, parametric, or non-parametric (according to Shapiro–Wilk normality test result), with Welch’s or Mann–Whitney’s correction. All results are presented as mean ± SEM and *p* values <0.05 were considered significant.

## Results

### Inosine attenuated astrocyte reactivity, preserved oligodendrocytes, reduced the number of macrophages, and increased the number of A2A receptor after SCI

**FIG. 1.**
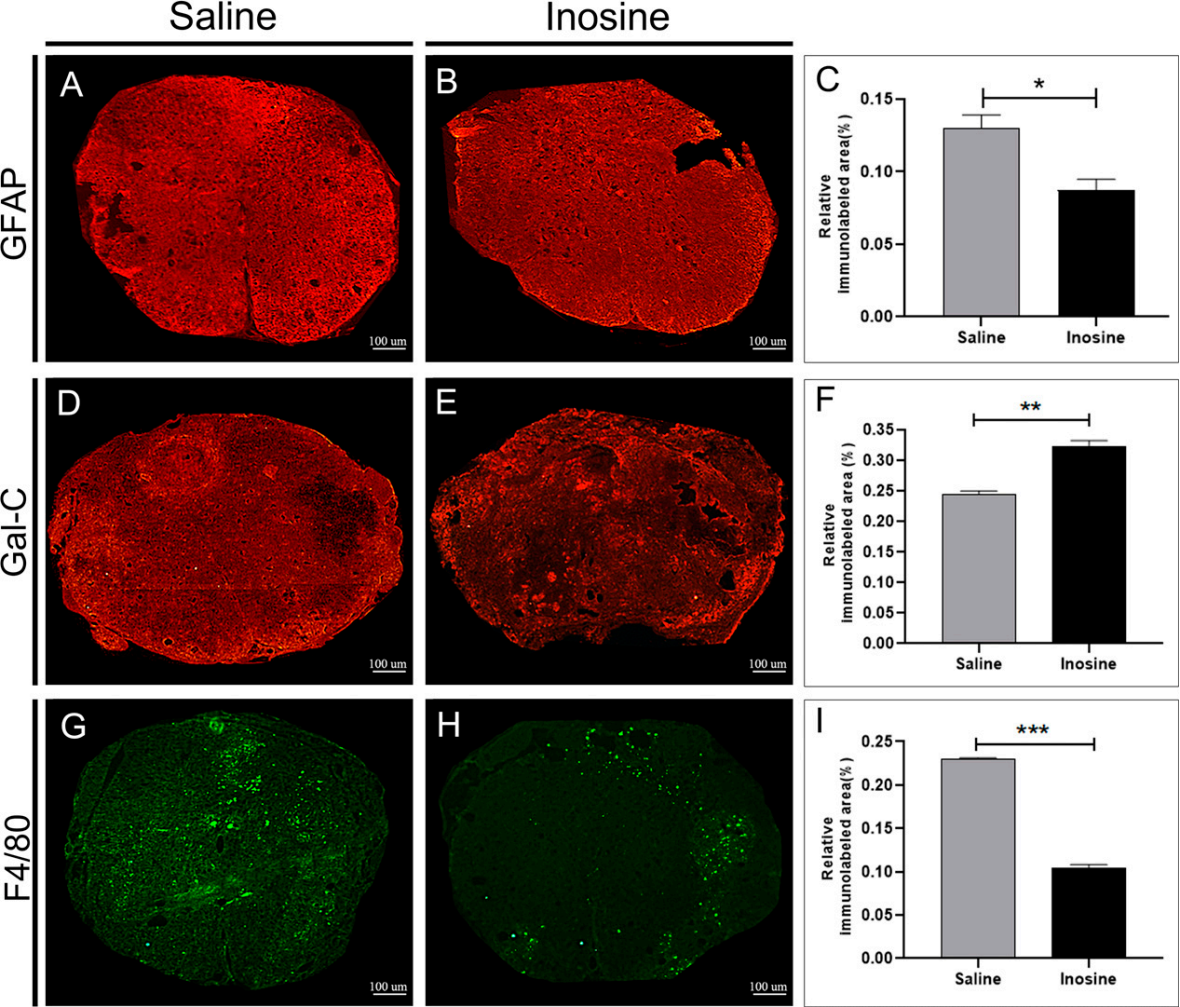
Relative immunolabeled area for GFAP, Gal-C, and F4/80. Note that the inosine group showed a reduced immunolabeled area for GFAP **(B)** and for F4/80 **(H)** when compared to the saline group **(A and G)**, as shown in graphs **(C and I)**. Regarding Gal-C, the inosine group **(E)**, presented a higher immunolabeled area than the saline group **(D)**, as shown in graph **(F)**. *p < 0.05; **p < 0.01; ***p < 0.001; scale bar = 100 µm. GFAP, glial fibrillary acidic protein.

To analyze the effects of inosine on astrocytes, oligodendrocytes, and macrophages, on rostral and caudal spinal cross-sections, we quantified the relative immunolabeled area of GFAP, Gal-C, and F4/80. The inosine group showed a significantly reduced immunolabeled area for GFAP (0.0874 ± 0.007327; *p < 0.05) when compared to the saline group (0.1302 ± 0.008944; Fig. 1A–C). Regarding the immunolabeled area for Gal-C, the inosine group showed a significantly larger immunolabeled area (0.3238 ± 0.008281; **p < 0.01), when compared to the saline group (0.2328 ± 0.01258; Fig. 1D–F). Last, concerning the immunolabeled area for F4/80, the inosine group also showed a significantly reduced immunolabeled area (0.1048 ± 0.003287; ***p < 0.001), when compared to the saline groups (0.2307 ± 0.0006387; Fig. 1G–I).

**FIG. 2.**
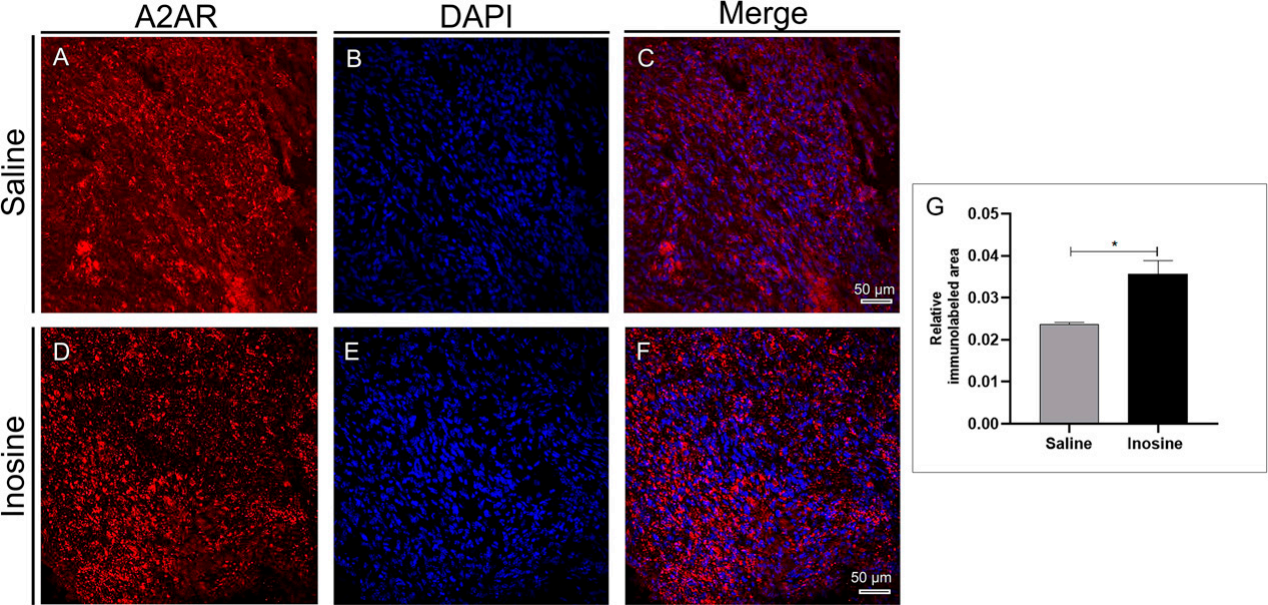
Relative immunolabeled area for A2AR. Note that the inosine group showed an increased immunolabeled area for A2A **(D–F)** when compared to the saline group **(A–C)**, as shown in graph **(G)**. We can also observe DAPI staining **(B and E)** and merge images **(C and F)**. *p < 0.05; Scale bar 50 µm.

To analyze the effects of inosine on A2A receptor expression on rostral and caudal spinal cross-sections, we quantified the relative immunolabeled area of A2AR. The inosine group (Fig. 2D–F and 2G) showed a significantly larger immunolabeled area for A2AR (0.03565 ± 0.0031; *p < 0.05) compared to the saline group (0.02364 ± 0.00048; Fig. 2A–C and G).

### Inosine increased the number of myelinated fibers

**FIG. 3.**
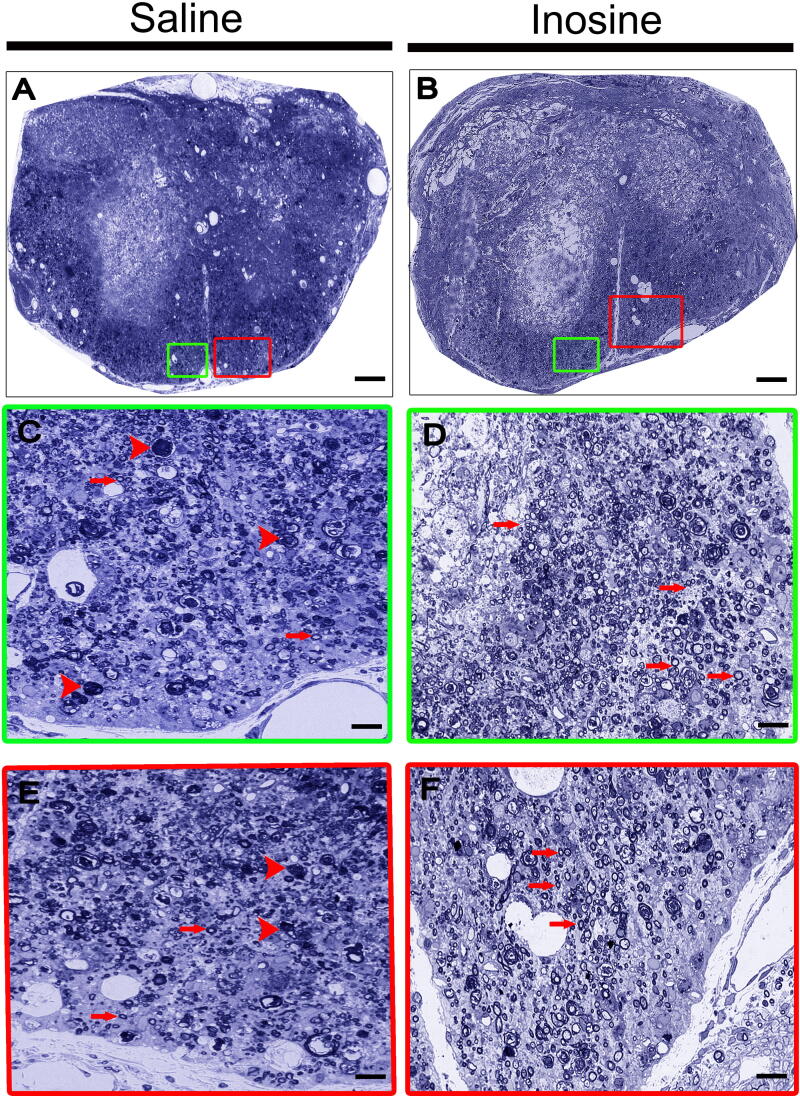
Semithin sections of spinal cords with saline **(A, C, and E)** and inosine **(B, D, and F)** groups. Note the tissue disorganization and some fibers under degeneration in saline (red arrow heads), while in the inosine group, there are many myelinated fibers (red arrows). Scale bar = 50 μm **(A and B)** and 10 μm **(C, D, E, and F)**.

To evaluate whether inosine promoted neuroprotection after SCI, we quantified the number of myelinated nerve fibers in the white matter using semithin sections of the lesion epicenter stained with toluidine blue (Fig. 3A–F). We observed that animals treated with inosine (Fig. 3B, D, and F) presented a better tissue organization compared to the saline group (Fig. 3A, C, and E). Observe several myelinated axons in the ventral white matter in the spinal cord of inosine animals, especially in higher magnification (Fig. 3D and F, red arrows). Another easily noticeable feature was the identification of many myelin debris even 8 weeks after the injury, suggesting that some fibers were still in the degeneration process (Fig. 3C and E, red arrowhead).

**FIG. 4.**
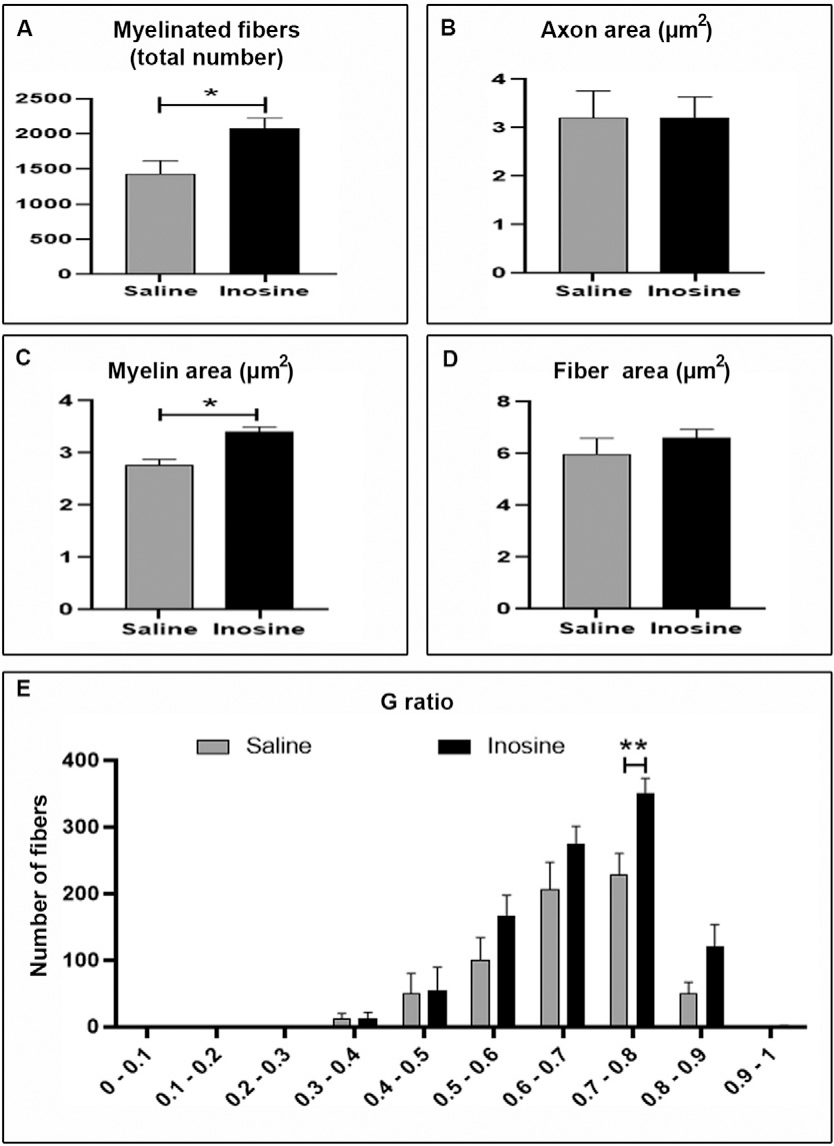
Morphometric analyses of myelinated fibers in semithin sections, stained with toluidine blue. The inosine group had more myelinated fibers than animals that received saline **(A)**. Note that inosine animals presented a larger myelin area when compared to saline **(C)**; however, no difference was observed in axon and fiber areas **(B and D)**. Last, observe that the inosine group has more fibers in the optimal *g*-ratio range (0.7–0.8) compared to saline animals **(E)**. *p < 0.05; **p < 0.01.

Quantitatively, animals treated with inosine showed more myelinated fibers (2081 ± 145.4; *p < 0.05) when compared to the saline group (1425 ± 192.8; Fig. 4A). In addition to the number of myelinated fibers, we quantified the areas of axons, myelin, and fibers and obtained the *g*-ratio values to evaluate the myelination after SCI (Fig. 4B–E). The saline group presented a reduced myelin area (2.759 ± 0.1147, *p < 0.05) when compared to the inosine group (3.398 ± 0.09727; Fig. 3C). However, there was no difference between the axon and fiber areas between groups (Fig. 4B and D). Although we did not observe any difference in axon and fiber areas between groups, the *g*-ratio showed a higher number of fibers in the optimal *g*-ratio range (0.7–0.8) in inosine-treated animals (350.7 ± 22.34; **p < 0.01) when compared to saline (227.3 ± 33.32; Fig. 4E), which correlates with better conduction velocity.

### Inosine promoted regeneration after injury

**FIG. 5.**
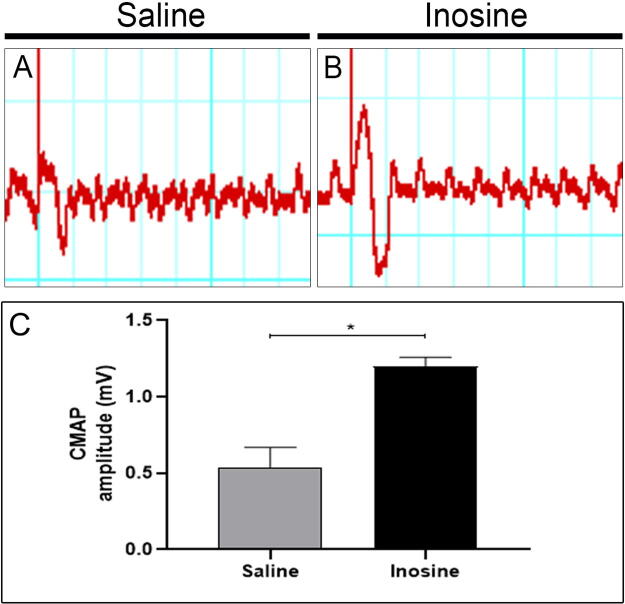
Analysis of the electroneuromyography. Wave register of CMAP amplitudes of saline **(A)** and inosine **(B)**. Note that the inosine group presented an increase in CMAP amplitude compared to animals treated with saline **(C)**. *p < 0.05. CMAP, compound muscle action potential.

To evaluate whether inosine can improve functional nerve regeneration after SCI, we performed the ENMG eight weeks after treatment to analyze the amplitude (mV) of the CMAP (Fig. 5A and B). We observed that the amplitude of CMAP was higher in the inosine-treated group (1.153 ± 0.02603; *p < 0.05) compared to the saline group (0.6100 ± 0.1153; Fig. 5C), suggesting that inosine promoted regeneration after SCI.

### Inosine improved motor and sensitive functions after SCI

**FIG. 6.**
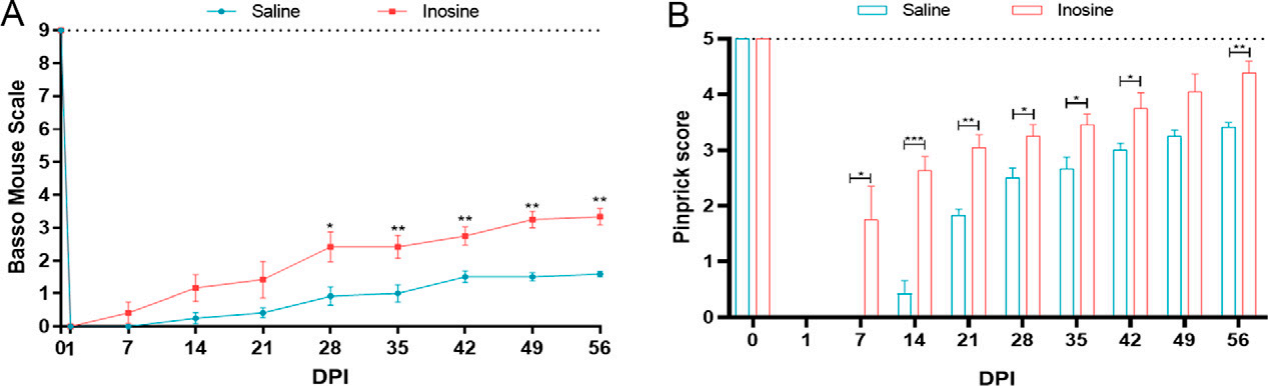
Behavioral assessment after SCI. **(A)** BMS score. Note that inosine animals reached the intermediate phase, and saline animals only reached the initial phase. **(B)** Pinprick score. Note that inosine had an early recovery of nociception and reached higher scores compared to saline animals. *p < 0.05; **p < 0.01. BMS, Basso Mouse Scale; SCI, spinal cord injury.

To evaluate whether inosine can improve motor and sensitive functions, we performed behavioral tests up to 8 weeks after treatment. At 24 h after surgery, both groups presented total paralysis (BMS = 0), with up to 7 days post-injury (DPI) in the saline group. However, some animals in the inosine group reached the initial phase of recovery, at 7 DPI. The saline-treated group reached the maximum score of 1.5. However, the group treated with inosine showed enhanced Basso, Beattie, and Bresnahan (BBB) scores at 14 DPI and continued to show improvement up to 49 DPI, reaching the maximum score. The animals maintained this maximum score at 56 DPI, indicating their ability to exhibit paw plantar placing with or without weight support, as well as occasional, frequent, or consistent dorsal stepping^28^ (Fig. 6A).

Regarding nociception, which was evaluated by the pinprick test, neither group presented a response at 24 h after surgery. However, at seven DPI, the inosine-treated group progressively improved the nociception, reaching the maximum score of 4 at 56 DPI, indicating that these animals recovered nociception in at least four areas of the paw. However, the saline-treated group reached 3 as the maximum score (Fig. 6B).

## Discussion

Despite scientific advances and existing therapeutic possibilities, no treatment has yet led to a successful functional recovery after SCI.^31^ Considering the limits of the available therapeutic approaches and the urgent need to develop new therapeutic strategies that can improve the regeneration of injured spinal cord tracts, we carried out this study to investigate whether intraperitoneal injection of inosine 24 h after compressive SCI results in morphological and functional improvement.

Among the proposed treatments, molecular therapy has shown therapeutic potential for SCI repair. In this context, inosine has promising effects on neuroprotection, promoting regeneration and minimizing the secondary events that occur after injury.^5,24,25,32^

To evaluate the reactivity of astrocytes, we performed immunohistochemistry using GFAP labeling. We observed smaller immunolabeled areas in the inosine group compared to the saline group, suggesting that inosine attenuated glial scar. In a mouse SCI model, blocking the interaction of type I collagen with astrocytes prevented astrocytic scar formation and promoted functional recovery, revealing that inhibition of glial scar formation contributes to a more effective regeneration.^33^ Thus, our data suggest that the attenuation of GFAP-positive cells creates a favorable environment for regeneration and correlates with better functional recovery, as observed in our inosine-treated animals.

To assess the survival of oligodendrocytes, we conducted immunohistochemistry using Gal-C, a marker of mature oligodendrocytes. Our findings revealed a greater number of Gal-C positive cells in the white matter of the inosine-treated group compared to the saline group. Our data are in accordance with an *in vitro* study that extracted oligodendrocytes from the brain of newborn rats that were induced to chemical hypoxia. Cultures pre-treated with inosine had more viable oligodendrocytes, demonstrating that inosine had a protective effect on mature oligodendrocyte lineages.^34^ Considering that Gal-C is a marker of mature oligodendrocytes, we raise the hypothesis that inosine promoted the survival of these glial cells after SCI, showing that this molecule was able to induce neuroprotection.

SCI causes prolonged inflammatory responses due to activation of innate immune responses that contribute to secondary injury.^35^ Macrophage recruitment presents a crucial role in inflammatory responses triggered after SCI and contributes to neuronal degeneration and regeneration.^36,37^ We evaluated the inflammatory microenvironment after SCI with F4/80, a specific marker of macrophages. Our data show that there were fewer macrophages in the inosine group, compared to the saline group. Inosine treatment significantly reduced the volume fraction of the ED-1+ (a specific marker for cytoplasmic protein that is homologous to the macrophages’ CD68 molecule) both in white and gray matters after moderate SCI, attenuating cavitation and reducing their impact on axonal regeneration after SCI.^11^ These results agree with the results obtained in our work, after intraperitoneal injection of inosine.

Inosine is an agonist of the four adenosine receptors and exerts its effects by binding to them.^38^ In the CNS, the A2A receptor is particularly important. After injury, A2A can modulate microglial proliferation through the release of BDNF,^39^ reduce pro-inflammatory cytokines,^40,41^ and promote axonal growth by increasing the expression of neurotrophic factor.^42,43^ Given the multiple effects of A2A activation, we investigated its presence following inosine treatment. Despite the increase in A2A receptor after SCI,^44^ our results show a higher expression of A2A in the microenvironment of the inosine-treated group compared to the saline-treated group. Inactivation of the A2A receptor by knockout resulted in an increase in the GFAP marker compared to wild-type animals in a model of white matter injury, induced by chronic brain perfusion.^41^ A2A activation prevents oligodendrocyte loss, by reducing the proapoptotic MAPK JNK, which is increased in oligodendrocytes after SCI.^45^ These findings suggest that the attenuation of glial reactivity and the oligodendrocyte survival observed in our results may depend on A2A activation by inosine.

Regarding the impaired axonal regeneration following SCI, previous studies have shown that inosine presented promising effects.^22,23,25^ To assess whether inosine promoted regeneration in our model, we performed morphological assessment using semithin cross-sections from the epicenter of the lesion, stained by toluidine blue. We observed that inosine-treated animals showed a better tissue organization, a greater number of myelinated fibers, a higher myelin area, and higher number of fibers in the ideal range of *g*-ratio compared to the saline groups. Our data agree with previous research of spinal cord hemisection showing that inosine promoted axonal sprouting from the corticospinal tracts, identified by higher expression of the GAP43 protein in the growth cones of inosine-treated animals.^22^ Another work by the same group demonstrated that inosine promoted synaptic contacts between collateral branches of the corticospinal tract and more fibers from the long propriospinal interneurons that projected from the cervical region.^23^ Thus, it is plausible to suggest that inosine treatment promoted axonal regeneration following SCI.

To confirm that inosine promoted regeneration after SCI, we performed ENMG and analyzed the CMAP amplitude 8 weeks after injury. Our results show that inosine promoted an improvement in CMAP amplitude if compared with saline-treated animals. A similar result was observed in a recent work done by our group, using inosine after sciatic nerve injury. In the study, authors also observed an improvement in CMAP amplitude in the inosine-treated groups if compared to controls.^46^ Altogether, our data support the conclusion that inosine promoted regeneration after SCI that led to improved functional outcomes.

As for functional outcomes, we performed behavioral tests to analyze motor and sensitive functions. First, we assessed motor function using the BMS and observed that the inosine-treated group reached the intermediate phase of recovery in the BMS, compared to the saline-treated group, which remained in initial recovery. These data are in accordance with studies performed on rats, in which inosine-treated animals presented better results on BBB score.^23,25^ Last, we assessed the nociceptive sensitivity using the pinprick test. Our results also show that inosine improved sensitive function compared to the saline-treated animals. This recovery in motor performance and nociception is supported by the better morphological outcomes also observed in our treated animals.

## Conclusion

Mice submitted to compressive SCI and treated with inosine intraperitoneal administration showed improved nociceptive sensitivity and had a better motor performance. Furthermore, the administration of inosine demonstrated an enhancement of A2A receptor and significant reduction in astrocyte reactivity and macrophage infiltration at the site of injury, leading to a smaller formation of glial scar tissue. This favorable outcome created an environment that supported the regeneration of axons, as indicated by more oligodendrocytes and myelinated fibers. In addition, the superior findings from the morphological analyses were consistent with the results obtained from electroneuromyography data and behavioral tests, indicating improved functional performance. Altogether, our data show that inosine holds promise as a viable molecular therapy for spinal cord compressive injuries.

## Data Availability

The data presented in this study are available on request from the corresponding author.

## Abbreviations Used

A2AR: Adenosine 2A receptor
BMS: Basso Mouse Scale
CMAP: Compound muscle action potential
CNS: Central nervous system
CST: Cortico spinal tract
DAPI: 4′,6-diamidino-2-phenylindole
ENMG: Electroneuromyography
GAL-C: Galactocerebrosidase
GAP43: Growth associated protein 43
GFAP: Glial fibrillary acidic protein
JNK: cJun N-terminal kinase
LPSN: Long propriospinal interneurons
MAG: Myelin associated glycoprotein
MAPK: Mitogen activated protein kinase
NOGO: Neurite outgrowth inhibitory protein
OCT: Optimal cutting temperature
OMGp: Oligodendrocyte myelin glycoprotein
PFA: 4% paraformaldehyde
RhoA: Rho GTPase A
ROCK: Rho associated protein kinase
SCI: Spinal cord injury
